# Epithelial cell-derived cytokine TSLP activates regulatory T cells by enhancing fatty acid uptake

**DOI:** 10.1038/s41598-023-28987-1

**Published:** 2023-01-30

**Authors:** Tadamichi Kasuya, Shigeru Tanaka, Jun Tamura, Keishi Etori, Jumpei Shoda, Koto Hattori, Yusuke Endo, Masayuki Kitajima, Takahiro Kageyama, Taro Iwamoto, Masaya Yokota, Arifumi Iwata, Akira Suto, Kotaro Suzuki, Harumi Suzuki, Steven F. Ziegler, Hiroshi Nakajima

**Affiliations:** 1 Department of Allergy and Clinical Immunology, Graduate School of Medicine, Chiba University, 1-8-1 Inohana, Chiba City, 260-8670 Japan; 2 Laboratory of Medical Omics Research, Kazusa DNA Research Institute, Kamatari, Kisarazu City, 292-0818 Japan; 3 Department of Omics Medicine, Graduate School of Medicine, Chiba University, Inohana, Chiba City, 260-8670 Japan; 4 Department of Immunology and Pathology, Research Institute, National Center for Global Health and Medicine, Konodai, Ichikawa City, 272-516 Japan; 5 Graduate School of Medicine, and Institute for Advanced Academic Research, Chiba, Japan; 6 Immunology Program, Benaroya Research Institute, Seattle, WA 98101 USA; 7 Department of Immunology, University of Washington School of Medicine, Seattle, WA 98195 USA

**Keywords:** Inflammation, CD4-positive T cells

## Abstract

Epithelial cells control a variety of immune cells by secreting cytokines to maintain tissue homeostasis on mucosal surfaces. Regulatory T (Treg) cells are essential for immune homeostasis and for preventing tissue inflammation; however, the precise molecular mechanisms by which epithelial cell-derived cytokines function on Treg cells in the epithelial tissues are not well understood. Here, we show that peripheral Treg cells preferentially respond to thymic stromal lymphoprotein (TSLP). Although TSLP does not affect thymic Treg differentiation, TSLP receptor-deficient induced Treg cells derived from naïve CD4^+^ T cells are less activated in an adoptive transfer model of colitis. Mechanistically, TSLP activates induced Treg cells partially through mTORC1 activation and fatty acid uptake. Thus, TSLP modulates the activation status of induced Treg through the enhanced uptake of fatty acids to maintain homeostasis in the large intestine.

## Introduction

Intestinal epithelial cells are essential in maintaining tissue homeostasis and work as barriers against non-self-antigens such as food antigens and gut microbiome^1^. In addition to the physical barrier function, epithelial cells influence immune cell function. The intestinal environment is easily changed by diet, stress, and infection; therefore, mucosal immune cells must adapt to the altered intestinal microenvironment^2^. To help immune cells adapt to environmental changes, epithelial cells produce several cytokines, including interleukin (IL)-25, IL-33, and thymic stromal lymphoprotein (TSLP)^1,2^. Although these epithelial cell-derived cytokines are highly related to type 2 inflammation^2^, recent studies have revealed the crucial roles of these cytokines in other types of immune responses^3^.

Regulatory T (Treg) cells are an indispensable lymphocyte subset for immune homeostasis^4^. It has been reported that epithelial cell-derived cytokines have roles in the function of Treg cells, especially at the mucosal surfaces^5–15^. However, the precise mechanisms by which epithelial cell-derived cytokines affect Treg differentiation and function remain unclear. For example, a recent study showed that keratinocytes in Mi-2-deficient mice produce TSLP, supporting Treg cells to acquire immunosuppressive function in the skin^12^. Others reported that robust TSLP production in keratinocytes by MC903 (Vitamin D3 analog) topical treatment primes dendritic cells (DCs) to acquire tolerogenic phenotype and let naïve CD4^+^ T cells differentiate into Treg cells, and this process is independent of TSLP receptor (TSLPR) expression on Treg cells^13^. Both reports have demonstrated that TSLP functions as a factor that favors Treg differentiation and function. Still, one study claimed the direct effect of TSLP on Treg cells^12^, and another reported the indirect impact and denied the direct effect of TSLP on Treg cell differentiation^13^.

We assessed the expression of receptors for epithelial cell-derived cytokines in the colonic Treg cells and focused on the function of TSLP in in vitro-induced Treg cells. We also assessed the effect of TSLP on the differentiation and activation of Treg cells. Finally, we analyzed the mechanisms by which TSLP enhances the activation of in vitro-induced Treg cells to understand the physiological significance of TSLP in the homeostasis of the large intestine.

## Results

### Treg cells express functional TSLP receptors in colonic lamina propria

**Figure 1:**
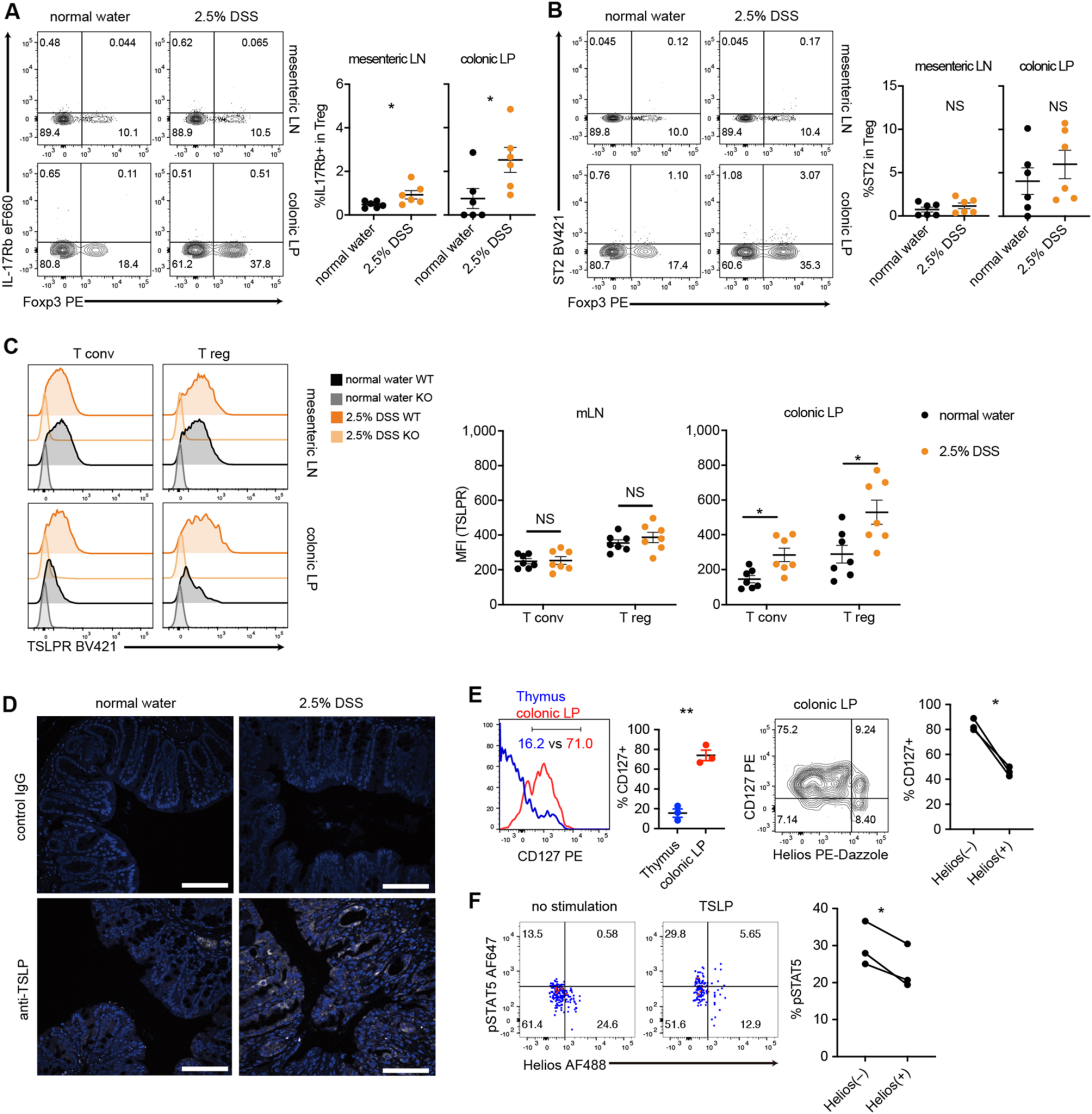
Functional TSLP receptor is expressed on Treg cells in mesenteric lymph nodes and colonic lamina propria. (**A**–**C**) The expression of receptors for epithelial cell-derived cytokines on wild-type (WT) CD4^+^ T cells in mesenteric lymph nodes (LN) and colonic lamina propria (LP) of mice given 2.5% dextran sodium sulfate (DSS) or normal water. Left, representative flow cytometry plot. Right, cumulative data expressed as mean ± SEM, n = 6–7 from at least 2 independent experiments. (**A**) IL-17Rb, (**B**) ST2, and (**C**) TSLPR were assessed. Cells from CD4^cre^TSLPR^f/f^ mice (KO) were negative controls in (**C**). *P < 0.05, **P < 0.01 as determined by unpaired t-test. NS: not significant. (**D**) The expression of TSLP in the colon of mice given 2.5% DSS or normal water. TSLP was expressed in white and DAPI in blue. Bars indicate 100 µm. Shown are representative of 2 independent experiments. (**E**) The expression of CD127 on Treg cells in the thymus and colonic LP. Representative histogram of CD127 (left, gated on Foxp3^+^ T cells) and CD127 vs. Helios (right, gated on Foxp3^+^ cells) and the cumulative data are presented. (**F**) Phosphorylation of STAT5 upon TSLP stimulation. Left, representative plots of phospho-STAT5 vs. Helios of Treg cells. Right, cumulative data, n = 3 from 3 independent experiments. *P < 0.05 as determined by paired t-test.

To determine the roles of epithelial cell-derived cytokines such as IL-25, IL-33, and TSLP in colonic Treg cells, we first analyzed the receptors for these cytokines on Treg cells in mesenteric lymph nodes and colonic lamina propria under the steady-state and inflammatory conditions. Treg cells expressing IL-17Rb, a component of IL-25 receptor, were almost absent in both mesenteric lymph nodes and colonic lamina propria (Fig. 1A). Although the percentage of IL-17Rb-expressing Treg cells was slightly elevated in mice treated with dextran sulfate sodium (DSS) water to induce colitis (DSS-induced colitis), the rate was only approximately 2% (Fig. 1A). As previously reported^5^, ST2-expressing Treg cells were present in colonic lamina propria; however, the percentage of ST2-expressing Treg cells was not significantly elevated when mice developed colitis (Fig. 1B). On the other hand, the levels of TSLPR (encoded by *Crlf2*), a component of TSLP receptor, on Treg cells and T conventional cells in colonic lamina propria were significantly elevated by DSS water treatment (Fig. 1C). Also, colonic epithelial cells expressed higher levels of TSLP when mice were given DSS water (Fig. 1D). These results suggest the roles of TSLP on Treg cells during colonic inflammation.

The functional TSLP receptor is composed of TSLPR and CD127 (also known as IL-7Ra), and numerous studies have shown that Treg cells do not express CD127^16–18^. Consistent with previous reports, most of Treg cells in the thymus were negative for CD127 (Fig. 1E). However, the majority of Treg cells in colonic lamina propria expressed CD127 (Fig. 1E). Intriguingly, almost all Helios-negative Treg cells, presumably peripheral Treg cells^19^, were positive for CD127 (Fig. 1E). Next, we sought to determine whether TSLP receptors expressed on Treg cells are functional. Treg cells harvested from colonic lamina propria (Supplementary Fig. 1) were stimulated with or without TSLP for 15 min, and the phosphorylation level of STAT5, a known downstream signaling molecule of the TSLP receptor in conventional T cells^20,21^ (Supplementary Fig. 2), was determined by flow cytometry. STAT5 was phosphorylated by TSLP stimulation in both Helios-negative and -positive Treg cells. Notably, Helios-negative Treg cells preferentially responded to TSLP stimulation (Fig. 1F). Together, these results support the idea that TSLP directly modulates Treg cell function and/or differentiation.

### TSLP is not essential for Treg cell differentiation

**Figure 2:**
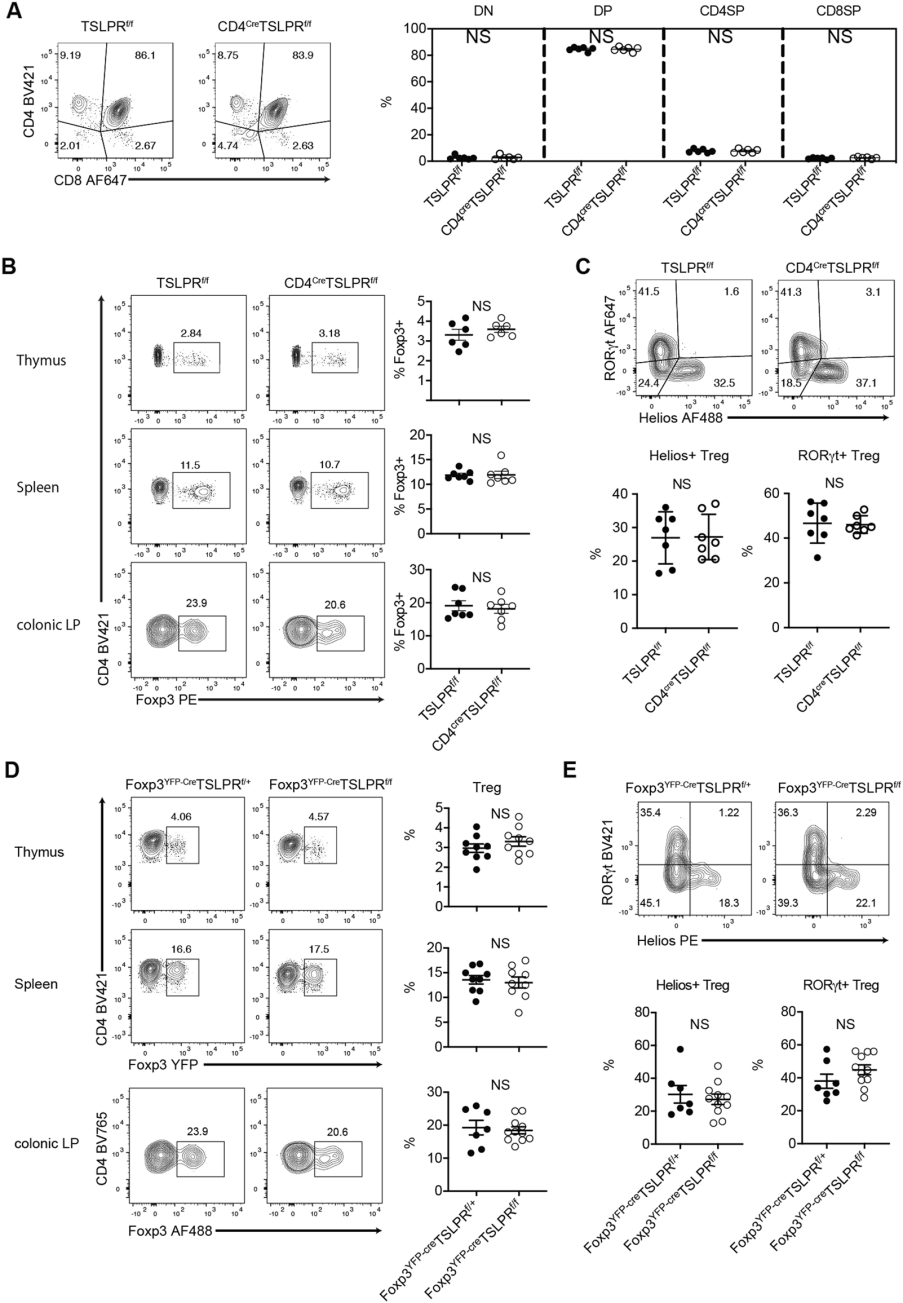
Treg cell differentiation in CD4^Cre^TSLPR^f/f^ and Foxp3^YFP-Cre^TSLPR^f/f^ mice. (**A**) Thymocyte development in TSLPR^f/f^ and CD4^Cre^TSLPR^f/f^ mice. Left, representative plots of CD4 vs. CD8. Right, cumulative data of CD4^−^CD8^−^ (double negative, DN), CD4^+^CD8^+^ (double positive, DP), CD4^+^CD8^−^ (CD4 single positive, CD4SP), and CD4^−^CD8^+^ (CD8 single positive, CD8SP). Data are expressed as mean ± SEM. n = 6 from 2 independent experiments. (**B**) Treg cell abundance in thymus, spleen, and colonic lamina propria (LP) of TSLPR^f/f^ and CD4^Cre^TSLPR^f/f^ mice. Left, representative FACS plots of Treg cells (gated on CD4^+^ T cells). Right, cumulative data expressed as mean ± SEM. n = 6 from 3 independent experiments. (**C**) Transcription factor expression in colonic LP Treg cells. Upper, representative plots of RORγt vs. Helios (gated on Foxp3^+^ cells). Lower, cumulative data expressed as mean ± SEM. n = 7 from 3 independent experiments. (**D**) Foxp3 expression in CD4^+^ T cells in Foxp3^YFP-Cre^TSLPR^+/f^ and Foxp3^YFP-Cre^TSLPR^f/f^ mice in thymus, spleen, and colonic LP. Representative FACS plot of Treg cells (gated on CD4^+^ T cells) and their cumulative data are shown. n = 9 from 3 independent experiments. (**E**) Transcription factor expression in colonic LP Treg cells. Upper, representative plots of RORγt vs. Helios (gated on Foxp3^+^ CD4^+^ cells). Lower, cumulative data expressed as mean ± SEM. n = 7–11 from 3 independent experiments.

Next, we analyzed the differentiation of Treg cells in T cell-specific TSLPR-deficient mice (CD4^Cre^TSLPR^f/f^) to address whether TSLP affects the differentiation of Treg cells. Consistent with the data obtained from systemic TSLPR-deficient mice (TSLPR^−/−^ mice)^22,23^, the composition of double negative cells, double positive cells, CD4 single positive cells, and CD8 single positive cells was not altered in CD4^Cre^TSLPR^f/f^ mice (Fig. 2A). The percentages of Treg cells in the thymus, spleen, and colonic lamina propria were comparable between CD4^Cre^TSLPR^f/f^ mice and littermate TSLPR^f/f^ mice (Fig. 2B). Helios expression in Treg cells was also analyzed since the functional TSLP receptors were mainly expressed on Helios-negative Treg cells (Fig. 1E). Still, Helios expression in colonic lamina propria Treg cells was not affected by the absence of TSLPR (Fig. 2C). We next analyzed RORγt expression in Treg cells as RORγt expression is a characteristic feature of colonic peripheral Treg cells^24^. Because activated STAT5 downregulates RORγt expression in T helper 17 cells^25^, we expected RORγt expression to be upregulated in colonic Treg cells of CD4^cre^TSLPR^f/f^ mice. However, contrary to our expectations, colonic lamina propria Treg cells in CD4^Cre^TSLPR^f/f^ mice displayed almost the same level of RORγt expression as Treg cells in control TSLPR^f/f^ mice (Fig. 2C and Supplementary Fig. 3A).

We also generated Foxp3^YFP-Cre^TSLPR^f/f^ mice in which only Treg cells lose TSLPR expression. Consistent with the findings of CD4^cre^TSLPR^f/f^ mice, there were no differences in the percentage of Treg cells in the thymus, spleen, or colonic lamina propria between Foxp3^YFP-Cre^TSLPR^f/f^ mice and littermate control Foxp3^YFP-Cre^TSLPR^f/+^ mice (Fig. 2D). Helios and RORγt expression in Treg cells in colonic lamina propria were also comparable between Foxp3^YFP-Cre^TSLPR^f/f^ mice and Foxp3^YFP-Cre^TSLPR^f/+^ mice (Fig. 2E and Supplementary Fig. 3B), suggesting that TSLPR deletion after Treg cell commitment does not affect Treg cell phenotype under the steady-state conditions. These results suggest that the lack of TSLPR does not influence the characteristics of Treg cells in lymphoid tissues and large intestines under steady-state conditions.

### TSLP enhances Treg cell activation both in vivo and in vitro

**Figure 3:**
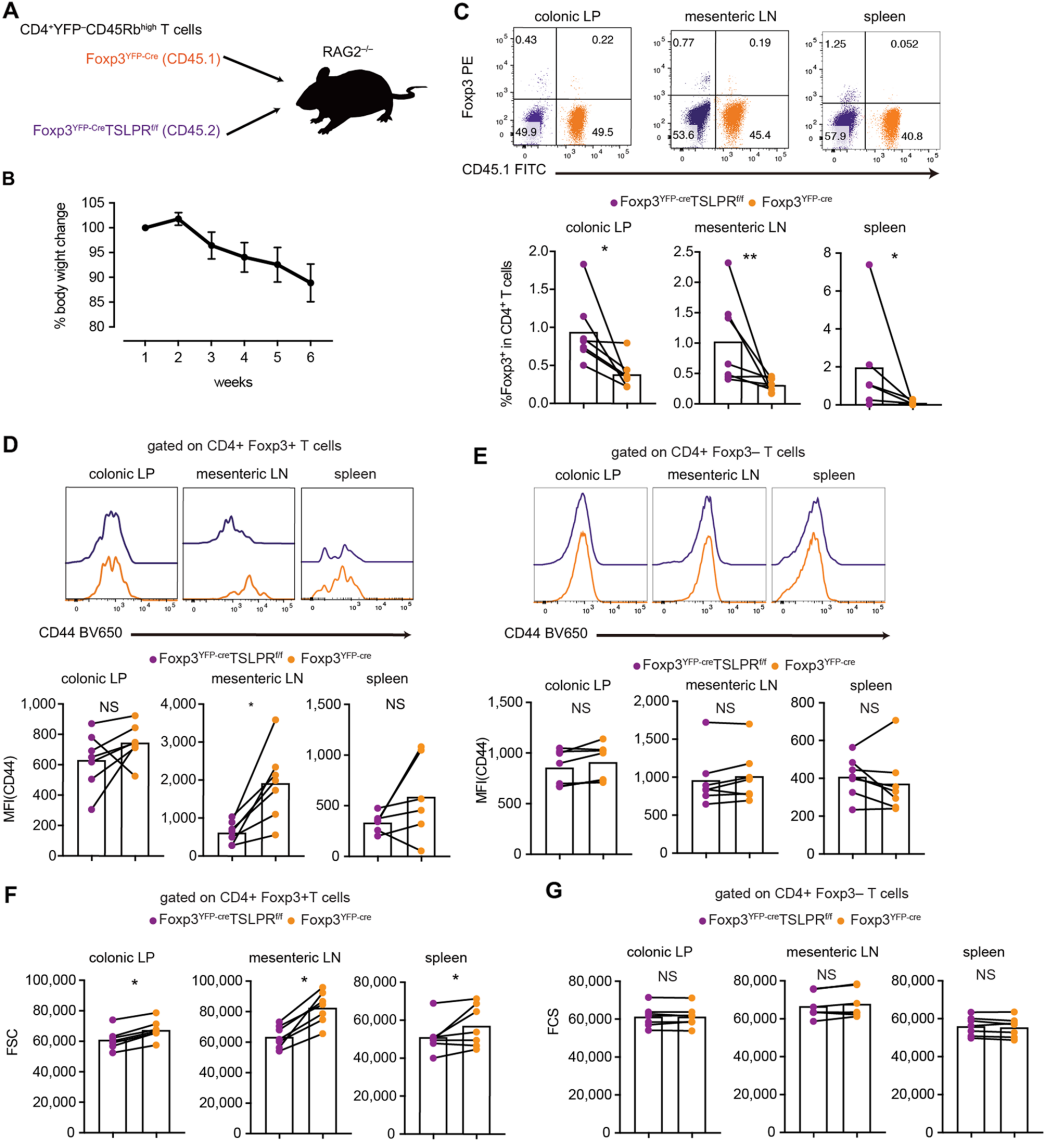
TSLP signaling enhances activation marker expression during experimental colitis in a competitive setting. (**A**–**G**) Adoptive transfer model of colitis. n = 7 from 2 independent experiments. (**A**) The same number of congenically marked naïve CD4^+^ T cells of Foxp3^YFP-Cre^ mice and Foxp3^YFP-Cre^TSLPR^f/f^ mice were injected into RAG2^−/−^ mice. (**B**) Bodyweight changes of the mice. (**C**) Representative FACS plots of Foxp3 vs. CD45.1 (gated on CD4^+^ T cells) in colonic LP, mLN, and spleen and cumulative data of CD4^+^ T cells expressing Foxp3. (**D**) CD44 expression on Treg cells in colonic LP, mLN, and spleen. Representative histograms of CD44 expression (gated on Treg cells) and their cumulative data. (**E**) CD44 expression on non-Treg cells in colonic LP, mLN, and spleen. Representative histograms of CD44 expression (gated on non-Treg cells) and their cumulative data. (**F**) Cell size (forward scatter [FSC]) of Treg cells of Foxp3^YFP-Cre^ mice and Foxp3^YFP-Cre^TSLPR^f/f^ mice in the adoptive transfer model of colitis. (**G**) Cell size (FSC) of non-Treg cells of Foxp3^YFP-Cre^ mice and Foxp3^YFP-Cre^TSLPR^f/f^ mice in the adoptive transfer model of colitis. *P < 0.05, and **P < 0.01 as determined by paired t-test.

TSLPR was preferentially expressed on peripheral Treg cells (Fig. 1F), and the differentiation of Treg cells in Foxp3^YFP-Cre^TSLPR^f/f^ mice under steady-state conditions was intact; we next investigated the role of TSLPR on peripheral Treg cells under inflammatory conditions. To assess this in the large intestine in a competitive setting, we co-transferred congenically marked naïve CD4^+^ T cells (CD4^+^YFP^−^ CD45Rb^high^ cells) from Foxp3^YFP-Cre^ mice (CD45.1) and Foxp3^YFP-Cre^TSLPR^f/f^ mice (CD45.2) into RAG2^−/−^ mice to see the differentiation and activation of Treg cells (Fig. 3A). Using this model, we confirmed the enhanced expression of TSLP in the epithelial cells (Supplementary Fig. 4A) and the increased TSLPR expression on Treg cells (Supplementary Fig. 4B). The mice subjected to this experiment lost body weight over time and showed severe diarrhea, suggesting that these mice developed colitis (Fig. 3B). Consistent with the previously-reported in vitro data^26^, the frequency of Treg cells was higher in cells from Foxp3^YFP-Cre^TSLPR^f/f^ mice than in cells from control Foxp3^YFP-Cre^ mice (Fig. 3C). To clarify the mechanism underlying the increased Treg cell population in cells from Foxp3^YFP-Cre^TSLPR^f/f^ mice, a similar experiment using CD4^cre^TSLPR^f/f^ mice was performed (Supplementary Fig. 4C). Interestingly, there was a significant decrease in cells from CD4^cre^TSLPR^f/f^ mice, suggesting an essential role of TSLP in T cell homeostatic expansion (Supplementary Fig. 4D). On the other hand, the percentage of Treg cells in each genotype was not altered (Supplementary Fig. 4D). These results suggest that TSLP signaling during homeostatic expansion is probably related to the cell proliferation/maintenance of T cells but not to Treg differentiation.

We also examined the activation status of transferred T cells of Foxp3^YFP-cre^TSLPR^f/f^ mice. The expression of CD44, an activation marker, was significantly lower in Treg cells derived from Foxp3^YFP-Cre^TSLPR^f/f^ mice than those from Foxp3^YFP-Cre^ mice in mesenteric lymph nodes (Fig. 3D). In contrast, there was no remarkable difference in the expression of CD44 among conventional T cells (Fig. 3E). Because activated lymphocytes generally increase in size^27^, we examined the size of Treg cells in an adoptive transfer model of colitis. Intriguingly, the cell size of TSLPR-deficient Treg cells was smaller than TSLPR-sufficient Treg cells (Fig. 3F), whereas the cell size of conventional T cells was not altered (Fig. 3G). These results suggest that TSLP mediates cell size control and activation in Treg cells.

**Figure 4:**
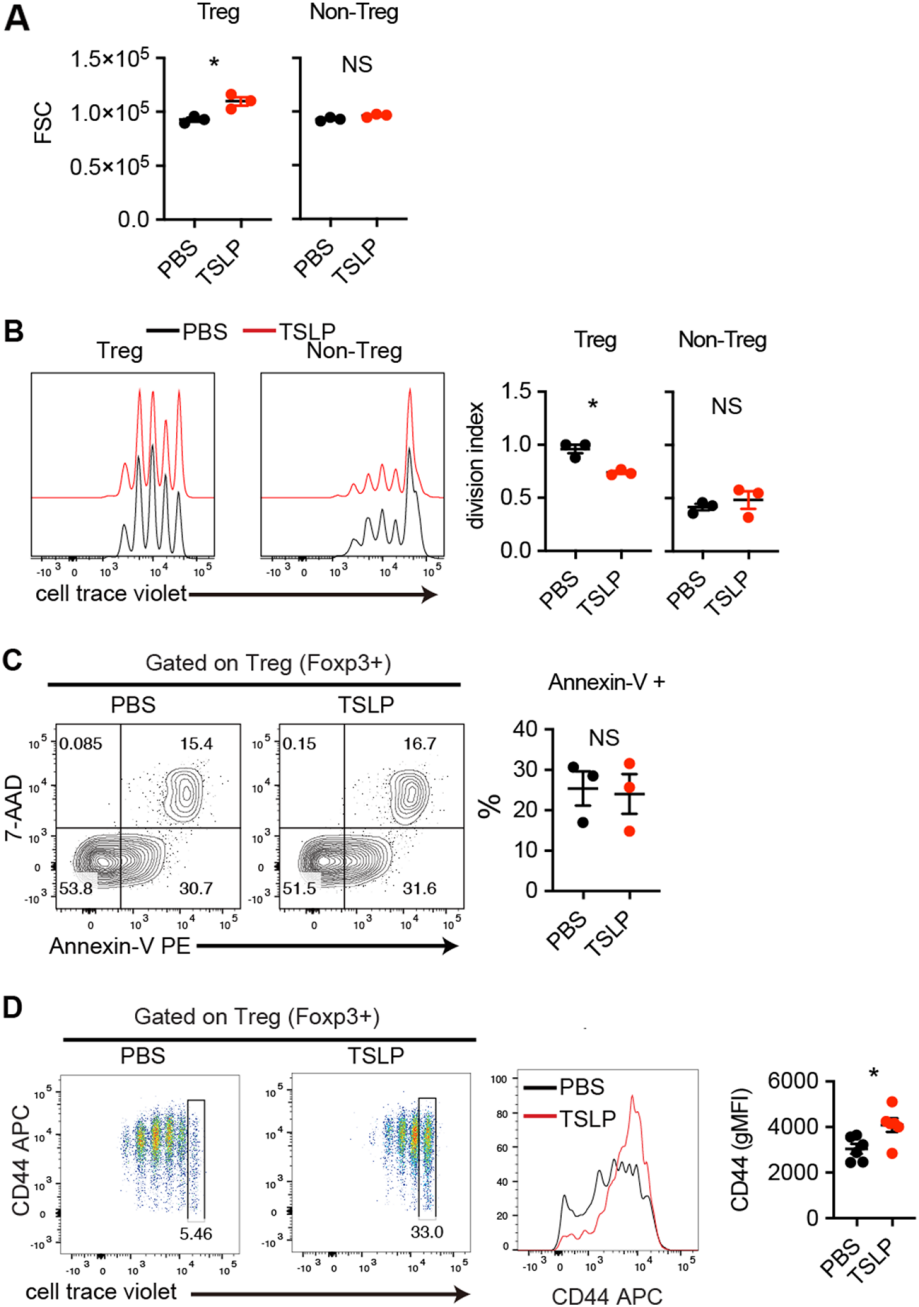
TSLP increases the size of Treg cells. (**A**–**D**) Cell size and cell division by TSLP. Naïve CD4^+^ T cells labeled with CellTrace Violet (CTV) were cultured under iTreg conditions for 3 days in the presence or absence of TSLP. The expression of Foxp3 defines Treg cells. Non-Treg cells are Foxp3^−^ cells in the same samples. n = 3 from 2 independent experiments. (**A**) Cell size determined by FSC of PBS- or TSLP-treated CD4^+^ T cells. (**B**) Left, representative histograms of CTV dilution. Right, cumulative data. (**C**) Apoptosis analysis. In vitro-induced Treg cells were stained with Annexin-V and 7-AAD. Left, representative FACS plots (gated on Treg cells). Right, cumulative data. n = 3 from 2 independent experiments. (**D**) Treg cell activation independent of cell proliferation. CTV-labeled cells cultured in the presence or absence of TSLP. Gated were the cells which did not proliferate and analyzed for the expression of CD44. n = 5 from 2 independent experiments. *P < 0.05 as determined by unpaired t-test.

To further address this issue, naïve CD4^+^ T cells were cultured under iTreg conditions in the presence or absence of TSLP for 3 days, and cell proliferation and cell size were analyzed. Consistent with the results of the adoptive transfer model of colitis, in vitro-induced Treg cells cultured with TSLP proliferated less efficiently and increased in size than control in vitro-induced Treg cells (Fig. 4A,B). In contrast, there was no difference in cell size of non-Treg cells (YFP^−^ cells) in the presence or absence of TSLP in the same culture well (Fig. 4A). To exclude the possibility that the disadvantage in cell proliferation in in vitro-induced Treg cells cultured with TSLP is just due to the induction of cell death, apoptosis was measured by Annexin-V staining. In vitro-induced Treg cells cultured with TSLP showed a comparable Annexin-V positive population to control in vitro-induced Treg cells (Fig. 4C).

To determine whether Treg activation by TSLP occurs independently of cell division, CTV-labeled Treg cells were cultured with or without TSLP, and the activation markers were examined for each cell division by flow cytometry. Interestingly, CD44 expression was elevated in the presence of TSLP even in Treg cells that did not undergo cell division (Fig. 4D). These results suggest that TSLP promotes Treg cell activation independent of cell proliferation.

### TSLP stimulation leads Treg cells to increase in size through the activation of mTORC1

**Figure 5:**
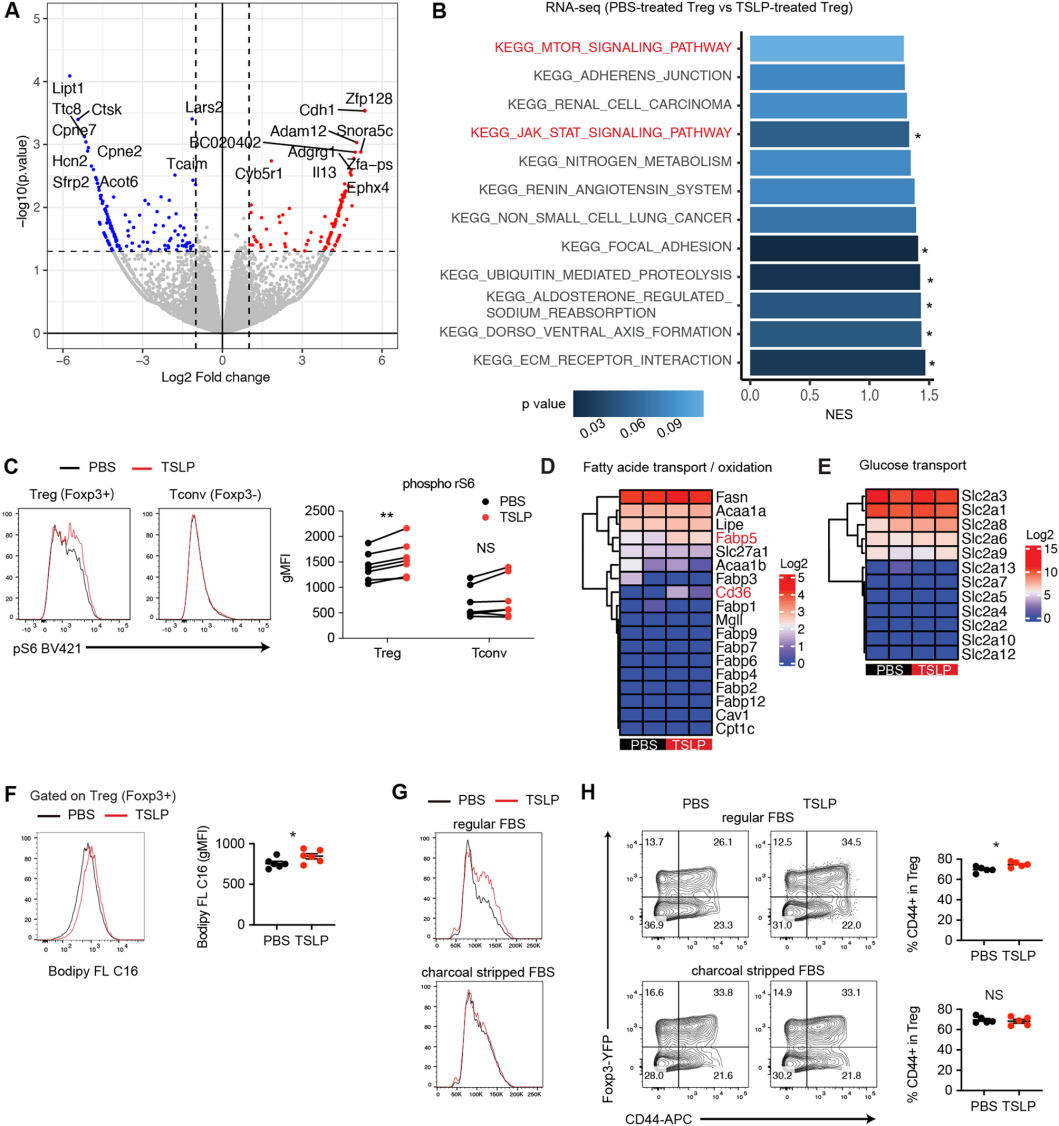
TSLP-stimulated in vitro-induced Treg cells are more dependent on fatty acids. (A) Differentially expressed genes of TSLP-stimulated Treg cells. RNA-seq of PBS- or TSLP-stimulated Treg cells were analyzed. Genes significantly upregulated by TSLP are in red and downregulated are in blue. Symbols of top 20 genes are also labeled. n = 2 for each condition. (**B**) Enriched KEGG pathway of TSLP-stimulated Treg cells. Pathways with P-values less than 0.1 were depicted. Pathways with P-value less than 0.05 were marked with *. (**C**) Phosphorylation of S6 by TSLP. Left, representative histograms. Right, cumulative data of geometric mean fluorescence intensity (gMFI). n = 7 from 4 independent experiments. *P < 0.05 as determined by paired t-test. (**D**,**E**) Heatmaps of fatty acid transport/oxidation-related genes (**D**) and of glucose transport-related genes (**E**) in TSLP-stimulated and PBS-stimulated Treg cells. (**F**) Fatty acid uptake by TSLP. Naive CD4^+^ T cells were cultured under iTreg conditions in the presence or absence of TSLP for 3 days. Cells were pulsed with Bodipy FL C16 for 1 h and subjected to flow cytometry. Representative histograms and cumulative data are shown. n = 6 from 2 independent experiments. (**G**–**H**) Treg cell activation by TSLP in a fatty acid-free medium. Naïve CD4^+^ T cells were cultured under iTreg conditions in the presence or absence of TSLP for 3 days in RPMI medium supplemented with regular FBS or charcoal dextran-treated FBS. Cell size (**G**) and CD44 expression (**H**) of Treg cells were analyzed by flow cytometry. Representative FACS plots (for FSC and CD44) and cumulative data (for CD44) are shown. n = 5 from 2 independent experiments. *P < 0.05 as determined by unpaired t-test.

It has been shown that TSLP signaling leads to STAT5 phosphorylation^20,21^. STAT5 is critical for cell proliferation; however, our data suggested that TSLP may negatively control cell proliferation. Thus, we speculated that TSLP stimulates signaling pathways other than JAK-STAT pathways. To assess this point, we performed a whole-transcriptome analysis. Treg cells were cultured for 3 h in the presence or absence of TSLP and subjected to RNA-seq analyses. The expression of several genes was altered by TSLP stimulation (Fig. 5A). As expected, KEGG-pathway analysis showed that the JAK-STAT signaling pathway was enriched in TSLP-stimulated Treg cells. In addition, mTOR signaling pathway was enriched, although the p value did not reach the statistical significance (p = 0.11) (Fig. 5B).

It has been shown that cell size but not cell proliferation is controlled by mTORC1 signaling^28^. Thus, we hypothesized that TSLP-mediated modification of Treg function is regulated by mTORC1 activation. To confirm TSLP-mediated mTORC activation, cells from mesenteric lymph nodes were stimulated in the presence or absence of TSLP for 5 min, and the level of phospho-S6, a well-known downstream target of mTORC1, was analyzed by flow cytometry. Consistent with previous reports^29,30^, Treg cells displayed a higher S6 phosphorylation level than conventional T cells (Fig. 5C). Importantly, TSLP induced S6 phosphorylation in Treg cells but not in conventional T cells (Fig. 5C). These results suggest that TSLP increases the size of Treg cells through the activation of mTORC1 pathways.

### TSLP enhances fatty acid uptake to maintain the activated phenotype in Treg cells

Recent studies have revealed that Treg cells preferentially utilize fatty acids rather than glucose^31^ and that fatty acid oxidation (FAO) is an essential metabolic process to acquire Treg cell suppressive function^32–35^. Considering that TSLP induced the activation of mTORC1 pathways and mTORC1 is an important regulator of cell metabolism, we hypothesized that TSLP changes the metabolic status of Treg cells. First, we analyzed fatty acid uptake because some fatty acid transporter-related genes (e.g., *Cd36* and *Fabp5*) were upregulated by TSLP stimulation (Fig. 5D,E and Supplementary Fig. 5). Naïve CD4^+^ T cells were cultured under iTreg conditions in the presence or absence of TSLP for 3 days, and then cells were pulsed with 1 µM bodipy FL C16 (palmitic acid) for 1 h. We found that the uptake of fluorescent-labeled palmitic acid was significantly elevated in TSLP-stimulated in vitro-induced Treg cells compared to that in control in vitro-induced Treg cells (Fig. 5F).

Next, we cultured in vitro-induced Treg cells in a medium supplemented with charcoal-stripped fetal bovine serum instead of regular bovine serum. Since charcoal dextran treatment efficiently removes fatty acids, this experiment allowed us to assess the role of fatty acids in TSLP-mediated cell growth and Treg activation. We found that upon TSLP stimulation, the size of in vitro-induced Treg cells was increased in the regular medium but not in the medium with charcoal-stripped FBS (Fig. 5G). We next analyzed the expression of CD44 on in vitro-induced Treg cells in the fatty acid-reduced conditions. We found that TSLP treatment significantly increased the expression of CD44 in the regular medium but not in the medium with charcoal-stripped FBS (Fig. 5H). These results suggest that TSLP-stimulated in vitro-induced Treg cells are more dependent on fatty acids.

## Discussion

In this study, we uncover the previously unknown roles of TSLP in regulating Treg cell function. We found that the majority of colonic peripheral Treg cells expressed functional TSLP receptors. The loss of TSLPR on T cells or Treg cells did not affect Treg cell differentiation, but TSLPR-deficient Treg cells decreased in size and expressed lower activation markers in an adoptive transfer model of colitis. Mechanistically, TSLP signaling increased cell size and enhanced fatty acid uptake in Treg cells. This cell metabolic status change might be beneficial in a fatty acid-rich environment such as the large intestine.

There is accumulating evidence suggesting Treg cells are fit to adapt to tissues where they are located. This event seems to be regulated by cells comprised of the tissues, such as epithelial cells and stromal cells^36^. As both epithelial cells and Treg cells maintain tissue homeostasis, it is plausible that these cells closely communicate with each other. Although it has been reported that IL-33 secreted from epithelial cells and immune cells is important for Treg cell-mediated immune responses in the tissues^5,9,37^, the role of TSLP in Treg cell function and differentiation is controversial. There are several reports showing TSLP promotes Treg cell differentiation and proliferation during tissue inflammation and infection^12,15,38^. On the other hand, Lei et al. have reported that TSLP interferes with antigen-specific Treg cell differentiation during allergic airway inflammation, breaking mucosal tolerance^26^. They transferred OVA-specific naïve CD4^+^ T cells (either TSLPR-sufficient or -deficient) to WT mice and sensitized them with OVA in the presence of TSLP. They found fewer OVA-specific Treg cells in lung draining lymph nodes of mice receiving TSLPR-deficient naïve CD4^+^ T cells, and TSLP-mediated decrease of Treg cells was cell-intrinsic. They also showed that Treg cell differentiation was diminished when cells were cultured with TSLP in vitro, thus concluding that TSLP disturbs antigen-specific Treg cell generation. Their study looked at the percentage of Foxp3^+^ cells in CD4^+^ T cell population, but they did not analyze Treg cell proliferation at a single-cell level. As we observed less proliferation of TSLP-conditioned Treg cells, it is possible that TSLP suppressed OVA-specific Treg cell proliferation after Treg cell commitment but did not hamper OVA-specific Treg cell differentiation during allergic airway inflammation. Another explanation would be that most of the data supporting TSLP’s positive role in Treg cell function were obtained from skin and intestine samples. So, it is also possible that TSLP regulates Treg cell function, differentiation, and proliferation in a site-specific or context-specific manner. Further studies are required to address this point.

Initially, it was shown that TSLP activates STAT5 in murine lymphocytes^39^, but unbiased RNA-sequence data of pathogenic T helper 2 (Th2) cells revealed that TSLP-mediated repression of Bcl-6 in Th2 cells is STAT5 independent^40^. Also, previous phospho-proteome analysis in a murine B cell line showed that TSLP mediates cell proliferation by activating several PI3 kinases, Src family kinases, and Btk in addition to STATs^41^. These results indicate that TSLP activates multiple signaling pathways in murine lymphocytes. We found that TSLP induced S6 phosphorylation, thus indicating the activation of mTORC1 signaling. Our current study is the first report showing that TSLP activates the mTORC1 pathway in murine CD4^+^ T cells, although it has been reported that TSLP promotes the phosphorylation of S6 and 4E-BP1 in pre-B cell lymphoma^42^. Importantly, mTORC1 activation by TSLP was dominantly observed in Treg cells (Fig. 5C), whereas STAT5 phosphorylation level was comparable between Treg cells and conventional T cells (Fig. 1F and Supplementary Fig. 2). This difference might be explained by the combination of signaling pathways, which each cell subset uses upon TSLP stimulation. Further study, such as unbiased phospho-protein analysis, is required; however, it is plausible that the molecular events under the TSLP receptor in each cell subset are highly diverse, and these benefits maintain homeostasis.

The roles of mTOR signaling in Treg cells are highly complicated. Early studies suggested that mTOR inhibition impairs Treg cell differentiation and expansion in vitro^43^ and in vivo^44^. However, Treg cells exhibit higher mTOR activation than conventional T cells (Fig. 5C)^29,30^, and mTOR activation is required for Treg function in vivo^30^. These findings suggest that mTOR signaling requirement in Treg cells depends on the model. A recent study showed that chronic mTOR inhibition resulted in inflammation and impaired Treg cell function in mucosal sites^45^. Mechanistically, mTOR activation in Treg cells is coupled with IRF4 induction and mitochondrial metabolism. They analyzed metabolites in Treg cells and found that activated effector Treg cells exhibit higher mitochondria-dependent energy production. In our study, TSLP-deficient Treg cells generated in RAG2^−/−^ mice expressed lower CD44 (Fig. 3D), and TSLP treatment upregulated CD44 expression in in vitro-induced Treg cells in a regular medium but not in a fatty acid-reduced medium (Fig. 5H). Also, TSLP stimulation enhanced the phosphorylation of mTORC1 (Fig. 5C). These results indicate that fatty acids are fueled to mitochondria to produce energy in TSLP-treated in vitro-induced Treg cells.

The abundance of fatty acids in the tissue may be important. A fascinating study shows the importance of dietary palmitic acid in IgA production^46^. They gave either palmitic acid-rich oil or regular oil to mice for 2 months and then measured the amounts of IgA and palmitic acid in the intestine and the serum. They found that the concentration of palmitic acid in the intestine was higher in mice fed on palmitic acid-rich oil than in mice fed on regular oil. On the other hand, there was no difference in the concentration of palmitic acid in the serum between the two groups. These findings suggest that fatty acids from the intestinal lumen affect the cells only in the intestine. So, it is reasonable that TSLP lets colonic Treg cells utilize fatty acids rather than glucose in the large intestine, especially during inflammatory conditions. It will be interesting to examine the abundance of metabolites used in Treg cells in various situations and tissues.

The effects of TSLP on the proliferation of Tregs and conventional T cells appear to be distinct. TSLPR deficiency in Treg cells had a positive effect on cell proliferation (Fig. 3C), while TSLPR deficiency in conventional T cells caused a significant decrease in cell expansion (Supplementary Fig. 4D). Possible explanations for this difference include the difference in the signaling pathways activated by TSLP. Our findings indicated that TSLP activates both STAT5 and mTOR in Treg cells, whereas TSLP activates STAT5 but not mTOR in conventional T cells (Fig. 5C and data not shown). We also consider the possibility that differences in the types of cytokines that affect cell division between Treg and conventional T cells may be involved. In particular, Treg cells require IL-2 for their differentiation and proliferation^47^, whereas conventional T cells are relatively less dependent on IL-2. Conventional T cells may be able to proliferate without IL-2 but with the stimulation of cytokines that activate STAT5, such as TSLP. These could explain the observed difference in the effect of TSLP on cell proliferation between Treg cells and conventional T cells in the adoptive transfer model. To our interest, there was no significant effect of Treg reduction by TSLP when naïve CD4^+^ T cells were cultured in the presence of TSLP from the beginning under iTreg conditions (Fig. 5H). Based on these data and the results of transfer experiments of CD4^cre^TSLPR^f/f^ mice into RAG2-deficient mice (Supplementary Fig. 4D), it is possible that the effect of TSLP on Treg reduction is more pronounced after Treg cell differentiation and is diminished by TSLP exposure prior to Treg cell differentiation.

In the current study, we did not investigate the suppressive function of TSLP-stimulated Treg cells. It should be determined whether TSLP-stimulated Treg cells that upregulate CD44 and cell size have superior suppressive function. Also, it would be interesting to analyze the suppressive function of TSLP-stimulated Treg cells under nutrient-restricted conditions. Another limitation is that we have not performed in vitro experiments on activation and fatty acid requirements using ex vivo Treg cells. Several studies have reported the differences between in vitro-induced Treg cells and ex vivo Treg cells^48^. Further studies are required to confirm the link between TSLP and Treg cell activation through fatty acid uptake, even in vivo situations. Overall, our current study revealed the importance of TSLP signaling in Treg cells in the large intestine. TSLP modulates the fatty acid utilization resulting in the activation of Treg cells. This study will help understand the relationship between epithelial cells and Treg cells, thus supporting the development of a novel therapeutic target for inflammatory diseases.

## Materials

### Mice

C57BL6/J (CD45.2) mice were purchased from Japan Clea (Tokyo, Japan). RAG2^−/−^ mice (008449), CD45.1 mice (002014), CD4^Cre^ mice (022071), and Foxp3^YFP-Cre^ mice (016959)^49^ were purchased from Jackson Laboratory (Bar Harbor, ME). TSLPR floxed mice were described previously^50^. Sex-matched mice aged 8–12 weeks were used. Mice were housed in specific pathogen-free facilities. All experiments were approved by the Animal Care and Use Committee at Chiba University (approval numbers: A4-53 and A4-58). All experiments were performed in accordance with relevant guidelines and regulations. The study was carried out in compliance with the ARRIVE guidelines. At the end of experiments, mice were expertly euthanized by cervical dislocation method for sample collection.

### Reagents

Anti-TSLP polyclonal antibody (cat# PA5-20321, RRID:AB_11156395), anti-IL-17Rb-eFluor660 (MUNC33, Cat# 50-7361-82, RRID:AB_2574289), anti-Foxp3-PE (Cat# 12-5773-82, RRID:AB_465936), -Alexa Fluor488 (Cat# 53-5773-82, RRID:AB_763537), or -Alexa Fluor700 (Cat# 56-5773-82, RRID:AB_1210557) (FJK-16 s), Bodipy FL C16, and Cell Trace Violet were purchased from Thermo Fisher Scientific (Waltham, MA). Anti-rabbit IgG-Alexa Fluor488 (Cat# 4412, RRID:AB_1904025) was from Cell Signaling Technology (Danvers, MA). Anti-CD4-V450 (Cat# 560468, RRID:AB_1645271, RM4-5), anti-pStat5-Alexa Fluor647 (47/Stat5, Cat# 612599, RRID:AB_399882), and anti-RORγt-Alexa Fluor647 (Cat# 562682, RRID:AB_2687546) or -BV421 (Cat# 562894, RRID:AB_2687545) (Q31-378) were from BD (San Jose, CA). Anti-CD3ε-PerCP (Cat# 100325, RRID:AB_893319) or -Alexa Fluor700 (Cat# 100216, RRID:AB_493697) (145-2C11), anti-CD4-APC-fire750 (Cat# 100568, RRID:AB_2629699) or -BV785 (Cat# 100552, RRID:AB_2563053) (RM4-5), anti-CD4-PE-Cy7 (Cat# 100422, RRID:AB_312707, GK1.5), anti-CD8-APC (Cat# 100711, RRID:AB_312750, 53–6.7), anti-CD45.1-Pacific Blue (Cat# 110722, RRID:AB_492866) or -FITC (Cat# 110706, RRID:AB_313495) (A20), anti-CD45.2-APC-Cy7 (Cat# 109824, RRID:AB_830789, 104), anti-CD127-PE (Cat# 135010, RRID:AB_1937251, A7R34), anti-CD44-BV650 (Cat# 103049, RRID:AB_2562600, IM7), -APC (Cat# 103012, RRID:AB_312963) or -PerCP-Cy5.5 (Cat# 103032, RRID:AB_2076204), anti-CD62L-PE (Cat# 104408, RRID:AB_313095, MEL-14), anti-ST2-BV421 (Cat# 145309, RRID:AB_2565634, DIH9), anti-TSLP-R-biotin (Cat# 151804, RRID:AB_2650790, 22H9), anti-Helios-PE (Cat# 137216, RRID:AB_10660749), -PE-Dazzle (Cat# 137232, RRID:AB_2565797), or -Alexa Fluor488 (Cat# 137223, RRID:AB_10661895) (22F6), anti-pS6-BV421 (Cat# 608610, RRID:AB_2814451, A17020B), Zombie aqua, Zombie NIR, streptavidin-BV421, and Annexin-V-PE were from BioLegend (San Diego, CA).

### Flow cytometry

For cytokine staining, cells were stimulated with phorbol 12-myristate 13-acetate (PMA)(20 ng/mL) and ionomycin (1 µg/mL) for 4 h in the presence of monensin (2 µM). After cell stimulation, cells were fixed and permeabilized with Fixation and Permeabilization Solution and Perm/Wash buffer (BD). Intracellular staining was conducted using Foxp3 intracellular staining reagents from Thermo Fisher Scientific. Phosphorylated Stat5 and ribosomal S6 were detected as previously described^51^. An apoptosis assay was performed as described elsewhere^51^. Flow cytometric analyses were performed on FACS Canto II, FACS LSR Fortessa X-20, or FACS LSR-II (BD) with FlowJo software (BD).

### DSS-induced colitis

Experimental colitis was induced with 2.5% DSS (MP Biomedicals, Irvine, CA) in the drinking water for 4 days, followed by normal water. Mice were weighed daily and sacrificed on Day 10. The colons were isolated, and the lamina propria cells were prepared as previously described^51^.

### Adoptive transfer model of colitis

An adoptive transfer model of colitis was performed as previously described^52^ with some modifications. Briefly, CD4^+^ T cells were magnetically sorted using a MojoSort Mouse CD4 T Cell Isolation Kit (BioLegend). CD4^+^ CD25^−^ CD45Rb^high^ cells were further sorted by an SH800 cell sorter (SONY, Tokyo, Japan) (Supplementary Fig. 6). Cells were injected intraperitoneally into RAG2^−/−^ mice (4 × 10^5^ cells/mice). Mice were weighed twice a week and sacrificed 4–6 weeks after the cell transfer. The colons and the mesenteric lymph nodes were collected and subjected to cell analysis.

### Cell culture

Naïve CD4^+^ T cells (CD4^+^ CD44^−^ CD62L^+^) were purified from the lymph nodes and the spleen with an SH800 cell sorter (Supplementary Fig. 7) following magnetic CD4^+^ T cell sorting and were rested in 1% BSA in PBS for at least 6 h. Cells were then cultured with plate-bound anti-CD3ε mAb (1 µg/mL) (Cat# 100363, RRID:AB_2632704, 2C11, BioLegend), anti-CD28 mAb (1 µg/mL) (Cat# 102102, RRID:AB_312867, 37.51, BioLegend), anti-IFNγ mAb (5 µg/mL) (Cat# 505802, RRID:AB_315396, XMG1.2, BioLegend), anti-IL-4 mAb (5 µg/mL) (Cat# 504102, RRID:AB_315316, 11B11, BioLegend), and recombinant human TGF-β (1 ng/mL) (R&D Systems, Minneapolis, MN) for 3 days (iTreg conditions). Where indicated, TSLP (100 ng/mL) (R&D Systems) or PBS was added to the culture medium. RPMI1640 medium (Sigma-Aldrich, St. Louis, MO) supplemented with charcoal dextran treated FBS (Cytiva, Marlborough, MA) were used in Fig. 5F,G.

### Immunohistochemistry

The formalin-fixed, paraffin-embedded samples were subjected to immunohistochemistry. For antigen retrieval, the slides were boiled in the citrate buffer for 5 min. After washing, the slides were blocked with 5% BSA overnight at 4˚C in a humid chamber. A primary antibody to TSLP or isotype control (10 µg/mL) was applied to the slides and incubated for 1 h at room temperature. The slides were then washed and incubated with anti-rabbit IgG-Alexa Fluor488 (5 µg/mL) for 1 h at room temperature in a dark place. Nuclear DNA was counterstained with DAPI, and the data were acquired with LSM710 (ZEISS, Oberkochen, Germany).

### RNA-seq analysis

Treg cells harvested from spleens and lymph nodes of Foxp3^YFP-Cre^ mice were subjected to the experiment (Supplementary Fig. 8). Sorted Treg cells were stimulated with or without 100 ng/mL TSLP for 3 h. Total RNA was purified with a Trizol reagent (Thermo Fisher Scientific). RNA-seq libraries were prepared using a NEBNext Ultra II Directional RNA Library Prep Kit for Illumina (NEB). Sequencing was performed on an Illumina HiSeq1500 (Illumina) in a 50-base single-end mode. Obtained reads were mapped on mm10 genome by Hisat2. HTSeq (v1.99.2) was used to obtain gene expression data. DESeq2 (v1.34.0) and clusterProfiler (v4.2.2) were used for further analysis. KEGG database^53^ was used for the enrichment analysis.

### Quantitative PCR analysis

The expressions of genes related to fatty acid transport were verified under the same experimental conditions as for RNA-seq analysis by quantitative PCR analysis. Total RNA was purified with a Trizol. cDNA was prepared using a SuperScript III (Thermo Fisher Scientific). The expression levels of *Cd36* and *Fabp5* were determined using an ABI StepOnePlus instrument (Applied Biosystems, Bedford, MA). PCR primers are as follows: Cd36 forward, 5’- GGACATTGAGATTCTTTTCCTCTG-3’; Cd36 reverse, 5’- GCAAAGGCATTGGCTGGAAGAAC-3’; Fabp5 forward, 5’-GACGACTGTGTTCTCTTGTAACC-3’; Fabp5 reverse, 5’- TGTTATCGTGCTCTCCTTCCCG-3’; β-actin forward, 5′-GCTCTGGCT CCTAGCACCAT-3′; β-actin reverse, 5′-GCCACCGATCCACACAGA GT-3′. The levels of target genes were normalized to the levels of β-actin.

### Statistical analysis

Data are presented as means with SEM. Prism 9 (GraphPad, San Diego, CA) was used for data analysis. Unpaired and paired t-tests were used to compare the two groups. Paired data were connected by a line. P values < 0.05 were considered statistically significant.

## Supplementary Information

Supplementary Figures.

## Data Availability

RNA-seq data have been deposited in Gene Expression Omnibus and are accessible through GSE189713. Any other data pertaining to the study will be made available on request.
