# Supplementary material for: Epithelial cell-derived cytokine TSLP activates regulatory T cells by enhancing fatty acid uptake
Source: Sci Rep. 2023 Jan 30;13:1653. doi: 10.1038/s41598-023-28987-1 (PMC9887060; doi:10.1038/s41598-023-28987-1)
Supplement: Supplementary file 1 — Supplementary Figures. [file 41598_2023_28987_MOESM1_ESM.docx]

**Supplementary Figures**

**
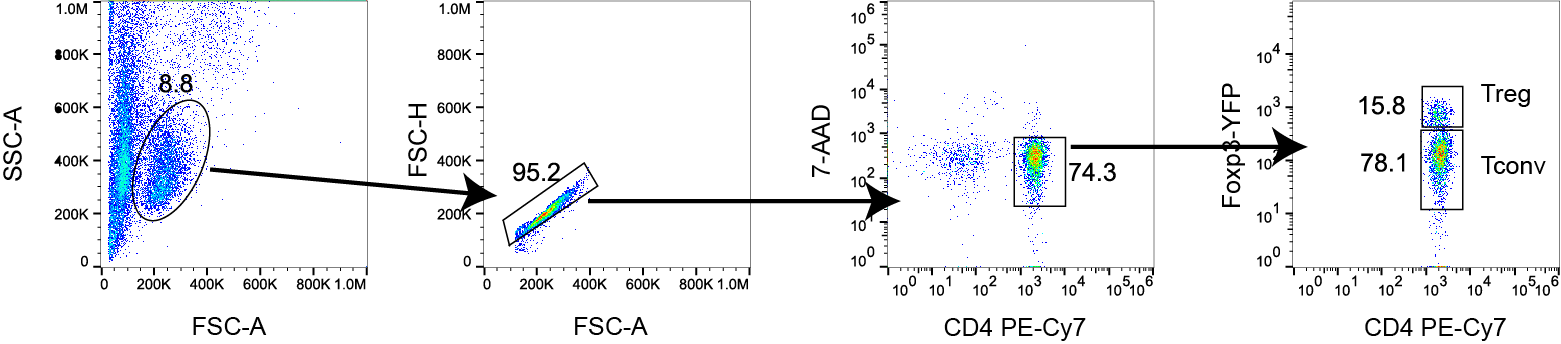
**

**Supplementary Figure 1. The gating to sort Treg and Tconv cells from colonic LP for phospho-STAT5 staining.**

Magnetically sorted cLP CD4^+^ T cells from Foxp3^YFP-cre^ mice were labeled with CD4-PE-Cy7 and 7-AAD. Cells were sorted using a cell sorter with this gating strategy for subsequent pSTAT5 staining.


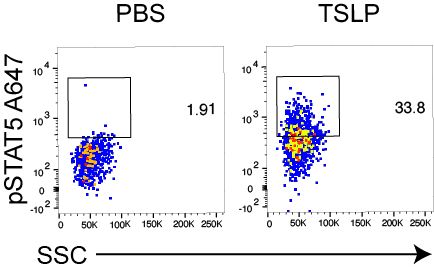


**Supplementary Figure 2. TSLP induces the phosphorylation of STAT5 in T conventional cells.**

Phosphorylation of STAT5 upon TSLP stimulation in T conventional cells. Representative FACS plots of phospho-STAT5 vs. SSC are shown.


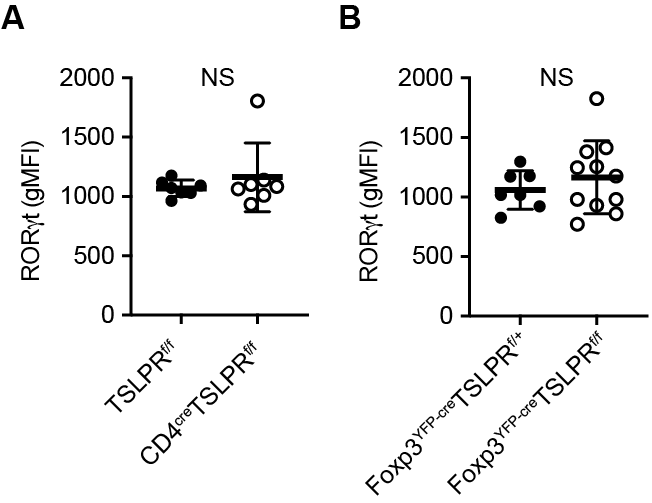


**Supplementary Figure 3. The levels of RORγt expression in RORγt^+^ Treg cells in colonic lamina propria.**

(**A**-**B**) Geometric MFI of RORγt in RORγt^+^ Treg cells in cLP in CD4^cre^TSLPR^f/f^ mice and control mice (**A**) and Foxp3^YFP-cre^TSLPR^f/f^ mice and control mice (**B**).


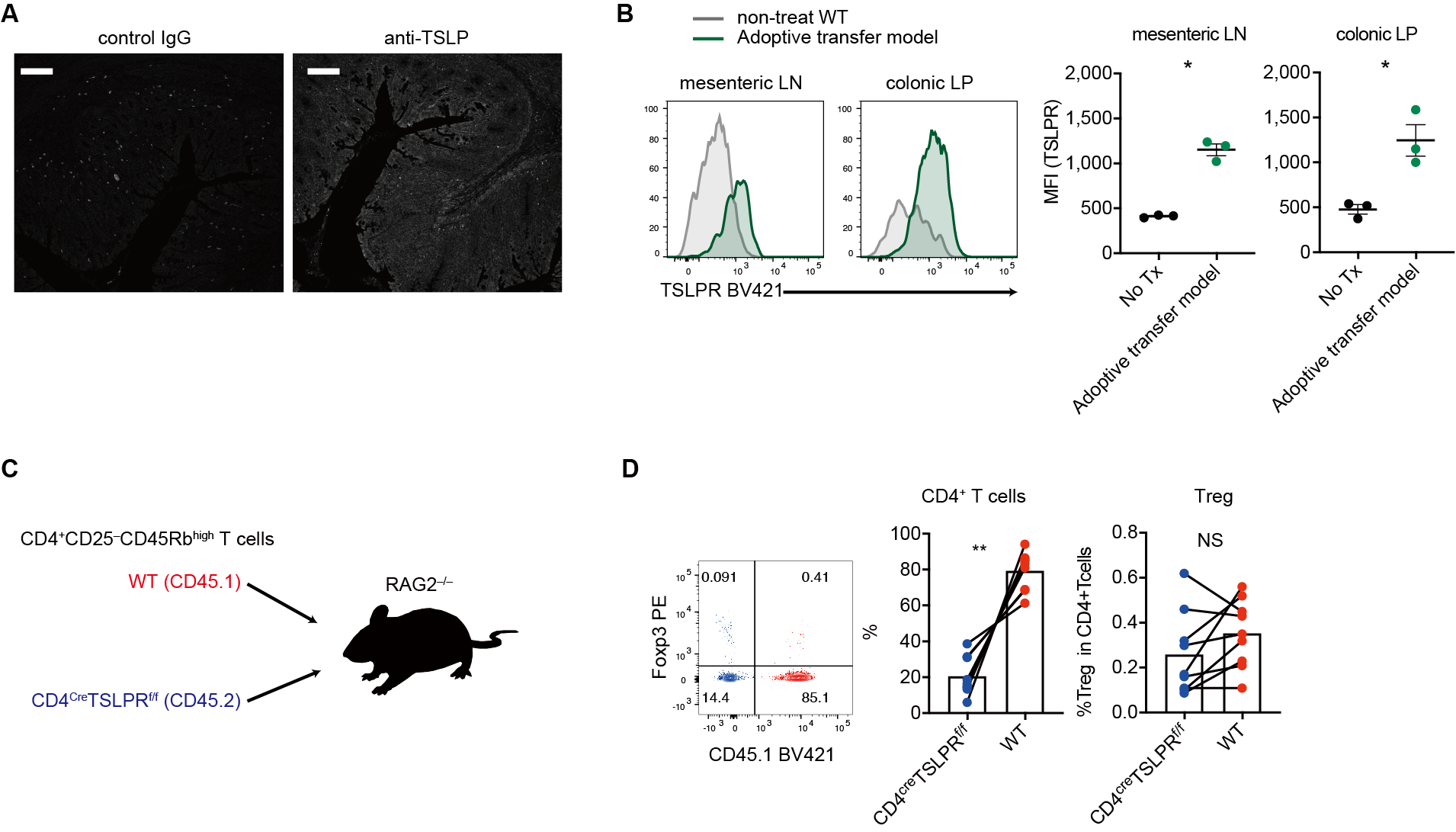


**Supplementary Figure 4. The related data in adoptive transfer model of colitis.**

**(A**) The expression of TSLP in the colon of RAG2^–/–^ mice receiving CD4^+^CD45Rb^high^ cells to develop an adoptive transfer model of colitis. TSLP was visualized in white. Bars indicate 100µm. Shown are representative of 2 independent experiments. (**B**) TSLPR expression on Treg cells in RAG2^–/–^ mice receiving CD4^+^CD45Rb^high^ cells or non-treated wild-type (WT) mice. Representative histograms of TSLPR expression on Treg cells in mesenteric lymph nodes and colonic LP and their cumulative data of MFI are shown. n = 3 from 2 independent experiments. * P < 0.05 as determined by unpaired t-test. (**C**) The same number of congenically marked naïve CD4^+^ T cells of WT mice (CD45.1) and CD4^Cre^TSLPR^f/f^ mice (CD45.2) were injected into RAG2^–/–^ mice. (**D**) Representative FACS plots of Foxp3 vs. CD45.1 (gated on CD4^+^ T cells) in colonic LP and cumulative data of CD4^+^ T cells and Treg cells. n = 9 from 2 independent experiments. ** P < 0.01 as determined by paired t-test.

**
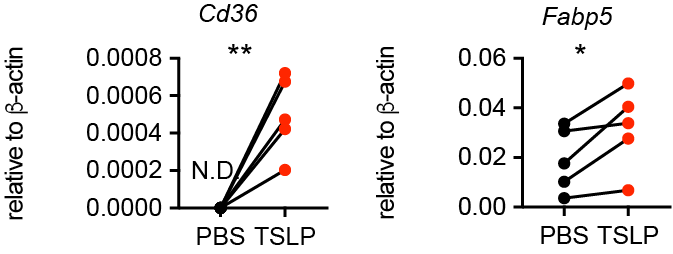
**

**Supplementary Figure 5. The expression of CD36 and FABP5 by TSLP stimulation in Treg cells.**

CD36 and FABP5 expressions were determined by quantitative PCR. Ex-vivo Treg cells were stimulated with 100 ng/mL TSLP or PBS for 3 hours and subjected to the analysis. The expressions were normalized to the levels of β-actin. n = 5 from 2 independent experiments. *P < 0.05, **P < 0.01 as determined by paired t-test.


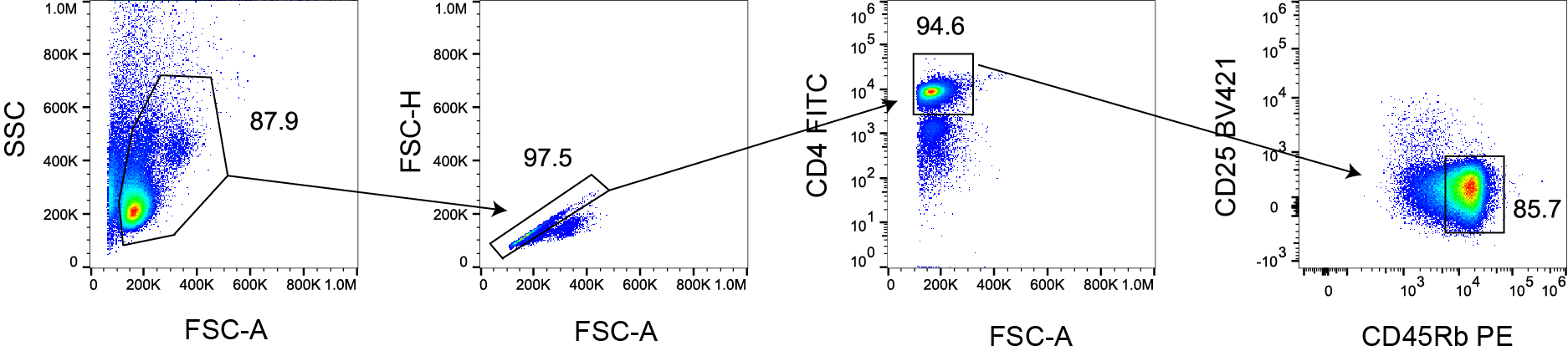


**Supplementary Figure 6. The gating to sort CD4^+^ CD45Rb^high^ naïve T cells from spleen.**

Magnetically sorted splenic CD4^+^ T cells were labeled with CD4-FITC, CD45Rb-PE, and CD25-BV421. Cells were then sorted using a cell sorter with this gating strategy.


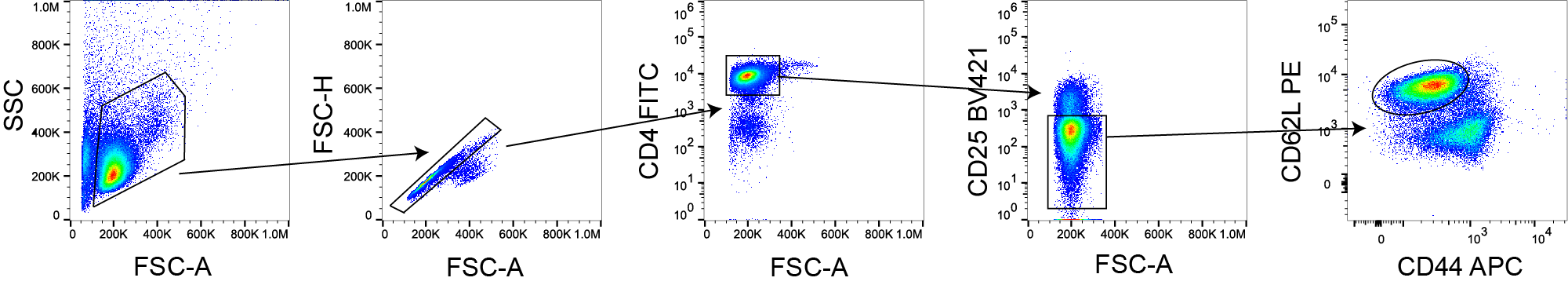


**Supplementary Figure 7. The gating to sort naïve CD4^+^ T cells for cell culture.**

Magnetically sorted splenic CD4^+^ T cells were labeled with CD4-FITC, CD25-BV421, CD62L-PE, and CD44-APC. Cells were then sorted using a cell sorter with this gating strategy.


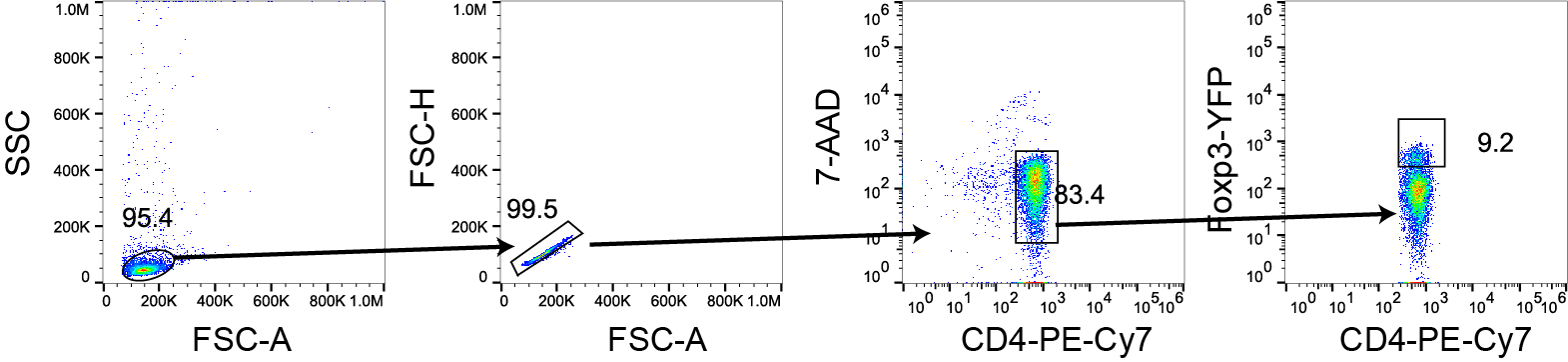


**Supplementary Figure 8. The gating to sort Treg cells from spleen and lymph nodes for gene expression analysis.**

Magnetically sorted CD4^+^ T cells from Foxp3^YFP-cre^ mice were labeled with CD4-PE-Cy7 and 7-AAD. Cells were sorted using a cell sorter with this gating strategy for RNA-seq and quantitative PCR analyses.
